# The gut microbiome of extremely preterm infants randomized to the early progression of enteral feeding

**DOI:** 10.1038/s41390-021-01831-w

**Published:** 2021-11-13

**Authors:** Ariel A. Salas, Kent A. Willis, Waldemar A. Carlo, Nengjun Yi, Li Zhang, William J. Van Der Pol, Noelle E. Younge, Elliot J. Lefkowitz, Charitharth V. Lal

**Affiliations:** 1 Division of Neonatology, Department of Pediatrics, School of Medicine, University of Alabama at Birmingham, Birmingham, AL USA; 2 Department of Biostatistics, School of Public Health, University of Alabama at Birmingham, Birmingham, AL USA; 3 Center for Clinical and Translational Science, University of Alabama at Birmingham, Birmingham, AL USA; 4 Department of Pediatrics, Duke University School of Medicine, Durham, NC USA; 5 Department of Microbiology, School of Medicine, University of Alabama at Birmingham, Birmingham, AL USA

## Abstract

**Background:**

Early progression of feeding could influence the development of the gut microbiome.

**Methods:**

We collected fecal samples from extremely preterm infants randomized to receive either early (feeding day 2) or delayed (feeding day 5) feeding progression. After study completion, we compared samples obtained at three different time points (week 1, week 2, and week 3) to determine longitudinal differences in specific taxa between the study groups using unadjusted and adjusted negative binomial and zero-inflated mixed models. Analyses were adjusted for a mode of delivery, breastmilk intake, and exposure to antibiotics.

**Results:**

We analyzed 137 fecal samples from 51 infants. In unadjusted and adjusted analyses, we did not observe an early transition to higher microbial diversity within samples (i.e., alpha diversity) or significant differences in microbial diversity between samples (i.e., beta diversity) in the early feeding group. Our longitudinal, single-taxon analysis found consistent differences in the genera *Lactococcus*, *Veillonella*, and *Bilophila* between groups.

**Conclusions:**

Differences in single-taxon analyses independent of the mode of delivery, exposure to antibiotics, and breastmilk feeding suggest potential benefits of early progression of enteral feeding volumes. However, this dietary intervention does not appear to increase the diversity of the gut microbiome in the first 28 days after birth.

**Trial Registration:**

ClinicalTrials.gov identifier: NCT02915549.

**Impact:**

- Early progression of enteral feeding volumes with human milk reduces the duration of parenteral nutrition and the need for central venous access among extremely preterm infants.
- Early progression of enteral feeding leads to single-taxon differences in longitudinal analyses of the gut microbiome, but it does not appear to increase the diversity of the gut microbiome in the first 28 days after birth.
- Randomization in enteral feeding trials creates appealing opportunities to evaluate the effects of human milk diets on the gut microbiome.

## Introduction

The past decade has seen considerable advances in our understanding of how the gut microbiome is assembled.^1^ Epidemiological studies in infants born at term have clearly linked disrupted development of the microbiome during the first several years of life to abnormal immune development and metabolic disease.^1^ More mechanistic studies, primarily in mice, have also begun to suggest how the disrupted assembly of the microbiome could lead to later disease.^2,3^

Recent translational studies indicate that the composition of the gut microbiome in infants born preterm follows a phased pattern of assembly.^4,5^ These studies also suggest that a delay in transitioning from low to high microbial diversity during these developmental phases is associated with slow weight gain. Enteral nutrition with human milk diets that include the provision of a variable number of bacteria and oligosaccharides may accelerate the transition between developmental phases of the gut microbiome, increase microbial diversity, and improve weight gain.^5,6^ These associations between the gut microbiome and favorable health outcomes likely involve direct effects on nutrient absorption and utilization and indirect effects on intestinal development, inflammation, and hormonal signaling.^7^

We have recently shown that early progression of enteral feeding volumes (i.e., within the first 96 h after birth) reduces the duration of parenteral nutrition and the need for central venous access among extremely preterm infants.^8^ However, the short-term effects of this early life dietary intervention on the gut microbiome have not been reported. We hypothesized that the early progression of enteral feeding volumes with human milk would increase microbial diversity in the gut microbiome of vulnerable preterm infants.

## Methods

### Study design

This study was a prespecified secondary analysis of the Early Progressive Feeding in Human Milk Fed Extremely Preterm Infants randomized clinical trial (ClinicalTrials.gov identifier: NCT02915549).^8^ All research procedures were carried out in accordance with the Declaration of Helsinki and under the supervision of the Institutional Review Board of the University of Alabama at Birmingham (UAB). Written informed consent was obtained from the parent/guardian of all infants enrolled in the study.

**Fig. 1:**
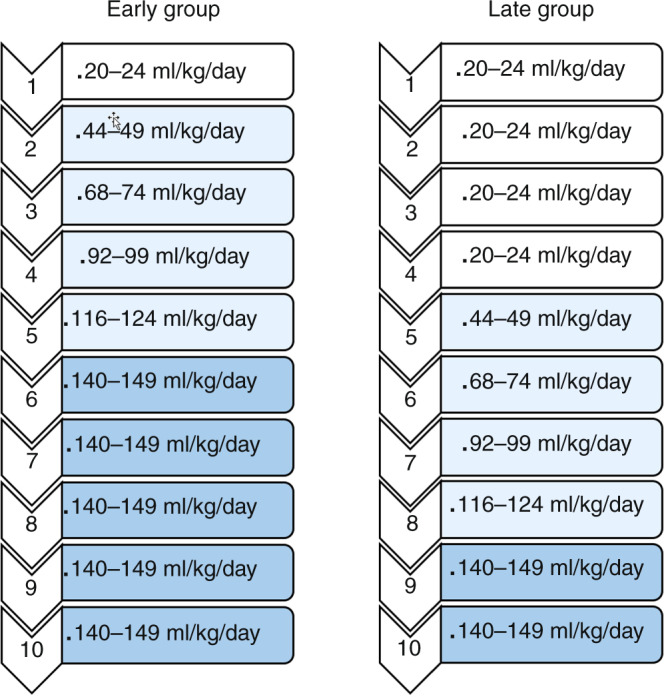
Feeding protocol to advance volumes according to feeding day. Feeding volumes were advanced on either feeding day 2 or feeding day 5. Rates of feeding advancement were similar in both groups.

Briefly, following computer-generated random-block sequences and using sequentially numbered, opaque, sealed envelopes, we randomized 60 extremely preterm infants with gestational ages 28 weeks or less to receive either early (feeding day 2) or delayed (feeding day 5) progression of enteral feeding volumes before or on feeding day 1 following a birthweight-based feeding protocol (Fig. 1), usually between 48 and 96 h after birth. Twin infants were randomized individually. The intervention was not masked.

During the trial, fecal samples from extremely preterm infants randomized to receive either early (i.e., feeding day 2) or delayed (i.e., feeding day 5) progression of enteral feeding volumes were collected from diapers immediately after study enrollment and then weekly until postnatal day 28 to determine the gut microbiome profile of study participants between postnatal days 5 and 7 at week 1, between postnatal days 12 and 14 at week 2, and between postnatal days 19 and 28 at week 3). Samples were stored frozen at −80C until 16S ribosomal DNA (rDNA) sequencing analysis was completed.

### Sample analysis

After study completion, genomic DNA was extracted from stored samples at the UAB University-Wide Microbiome Center. Once the DNA samples were prepared, PCR was used with unique bar-coded primers to amplify the V4 region of the 16S rDNA to create an amplicon library from individual samples using the Illumina MiSeq platform (Illumina Inc., San Diego, CA). The sequence data covered the 16S rRNA V4 region with a PCR product length of ~255 bases and 250 base paired-end reads.

### Bioinformatics

Raw sequence reads were aligned against the Greengenes database^9^ using a data analysis pipeline based primarily on QIIME v 1.9. Further data analysis was then carried out in R primarily utilizing the package *phyloseq.*^10^ For further data visualization, we also imported processed OTU tables and metadata into Calypso v8.84.^11^ We prespecified exclusion of any samples with a read count <1000. Alpha diversity was then quantified using the Shannon diversity indices and linear mixed models (LMMs) were used to test for differences between alpha diversity and host characteristics.^12^ We used permutational multivariate analysis of variance (PERMANOVA) to determine significant differences in beta diversity as quantified by the Bray–Curtis dissimilarity distance matrices. To relate differences in beta diversity between the hosts, we also used redundancy analysis (RDA).

### Longitudinal and single-taxon analysis

To identify specific taxa that changed over time and differed between the study groups, we utilized negative binomial and zero-inflated mixed models (NBMM),^13–16^ as implemented in the R package *NBZIMM* (https://github.com/nyiuab/NBZIMM). We included the total sequence reads as offset in the negative binomial mixed model to account for varying total sequence reads. We used the false discovery rate method to adjust for multiple hypothesis testing.

## Results

We collected 137 fecal samples from 51 of 60 extremely preterm infants randomized to receive early or delayed enteral feeding progression (78 fecal samples obtained from 23 infants randomized to receive early feeding progression and 59 fecal samples obtained from 28 infants randomized to receive delayed feeding progression) (Table 1). Fecal samples from nine infants who either died or developed SIP/NEC before postnatal day 14 were not analyzed (5 in the early group and 4 in the late group). Nearly 85% of participants (*n* = 38) included in the primary analysis had all three fecal samples collected at week 1, week 2, and week 3. The rest (*n* = 13) contributed to the analysis with either 1 or 2 fecal samples collected.

**Table 1:** Baseline characteristics.

	Early feeding group (*n* = 23)	Late feeding group (*n* = 28)	
Demographic characteristics			
Birth weight (g), mean ± SD	923 ± 268	798 ± 242	0.09
Gestational age (weeks), median (IQR)	27 (25–28)	26 (24–27)	0.16
Male, *n* (%)	9/23 (39)	8/28 (28)	0.42
Black race, *n* (%)	11/23 (48)	19/28 (68)	0.15
Exposure to a full course (two doses) of antenatal steroids, *n* (%)	15/23 (65)	15/28 (54)	0.40
Vaginal delivery, *n* (%)	13/23 (56)	16/28 (57)	0.96
Exposure to maternal antibiotics 72 h prior to delivery, *n* (%)	20/23 (87)	21/28 (75)	0.28
Days to regain birth weight, mean ± SD	18 ± 7	16 ± 6	0.37
Postnatal growth failure at 36 weeks^a^, *n* (%)	7/21 (33)	11/25 (44)	0.46
Exposure to antibiotics during the first 28 days after birth in days^b^, median (IQR)	3 (2–6)	4 (2–10)	0.32
Percentage of maternal milk intake^c^, median (IQR)	55 (20–100)	90 (28–100)	0.29
Duration of parenteral nutrition in days, median (IQR)	8 (5–11)	11 (9–16)	<0.001

^a^Postnatal growth failure was defined as weight-for-age <10th percentile.

^b^Postnatal exposure to antibiotics was defined as the total number of days in which antibiotics were administered during the first 28 days after birth.

^c^Percentage of maternal milk intake was measured over a 28-day period. The sum of the daily maternal milk volumes was divided by the total feeding volume administered during the first 28 days after birth.

### Alpha diversity

Alpha diversity quantifies the diversity within a sample. We developed two models to detect differences in alpha diversity between groups. When treating the study intervention as a fixed effect with a random intercept, neither a simple univariate model (*p* = 0.5126, LMM, model A) nor a more complex model with several covariates (i.e., mode of delivery, the proportion of breastmilk intake, exposure to postnatal antibiotics, race, sex, gestational age, and maternal exposure to antibiotics) found a significant difference in alpha diversity between groups (*p* = 0.4779, LMM, model B). The linear correlation between the Shannon diversity index and other metrics of alpha diversity was high (correlation coefficients >0.90)

### Beta diversity

**Fig. 2:**
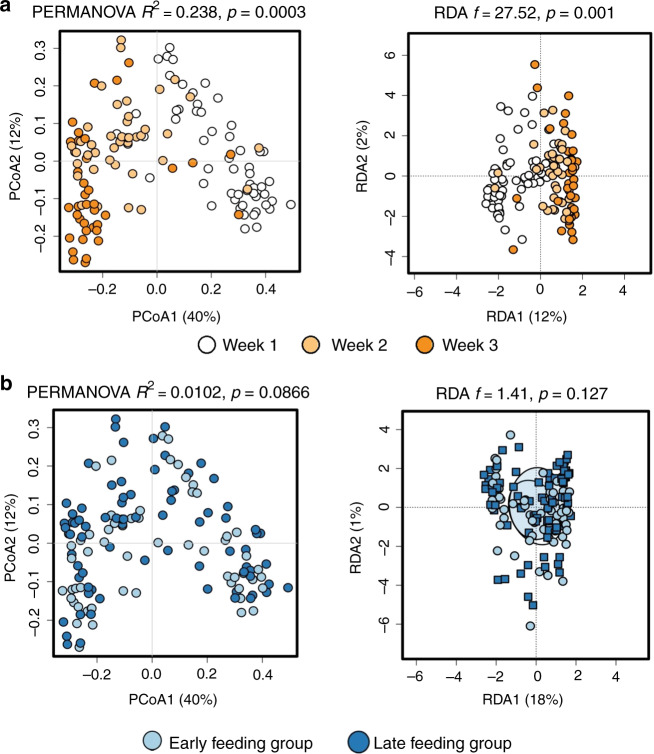
Differences in microbial diversity between groups. Differences in beta diversity according to advancing weeks of life were statistically significant in PERMANOVA and RDA analyses (**a**). Differences in beta diversity according to study group were not statistically significant by principal coordinate analysis (PCoA) of Bray–Curtis dissimilarity (PERMANOVA, *R*^2^ = 0.01, *p* = 0.09) or redundancy analysis (RDA, *f* = 1.41, *p* = 0.13). PERMANOVA permutational multivariate ANOVA, RDA redundancy analysis.

**Fig. 3:**
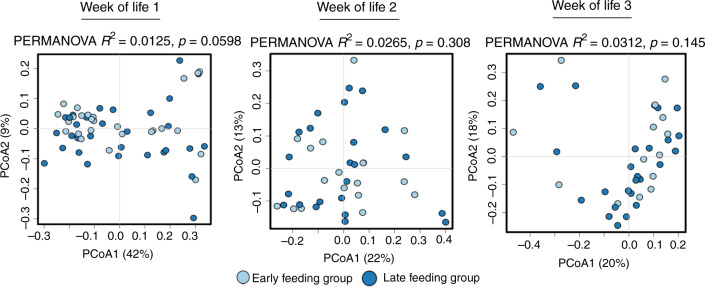
Differences in microbial diversity between groups by week of life. Significant differences in beta diversity between early and late feeding progression groups at three different time points (*n* = 57 at week 1, *n* = 42 at week 2, *n* = 38 at week 3) were not detected by principal coordinate analysis (PCoA) of Bray–Curtis dissimilarity. PERMANOVA permutational multivariate ANOVA.

Beta diversity is used to quantify differences in microbial diversity between groups. There were strong differences in beta diversity according to week of life (*p* = 0.0033, *R*^2^ = 0.238, PERMANOVA) (Fig. 2a). No significant differences in beta diversity between groups were found by either PERMANOVA or RDA (Fig. 2b). Due to the magnitude of the differences observed with each progressive week of life, the analysis by week of life and the resulting six comparison cohorts are shown in Fig. 3a, b. No significant differences in microbial community composition were apparent with the early progression of enteral feeding volumes.

### Longitudinal and single-taxon analysis

**Fig. 4:**
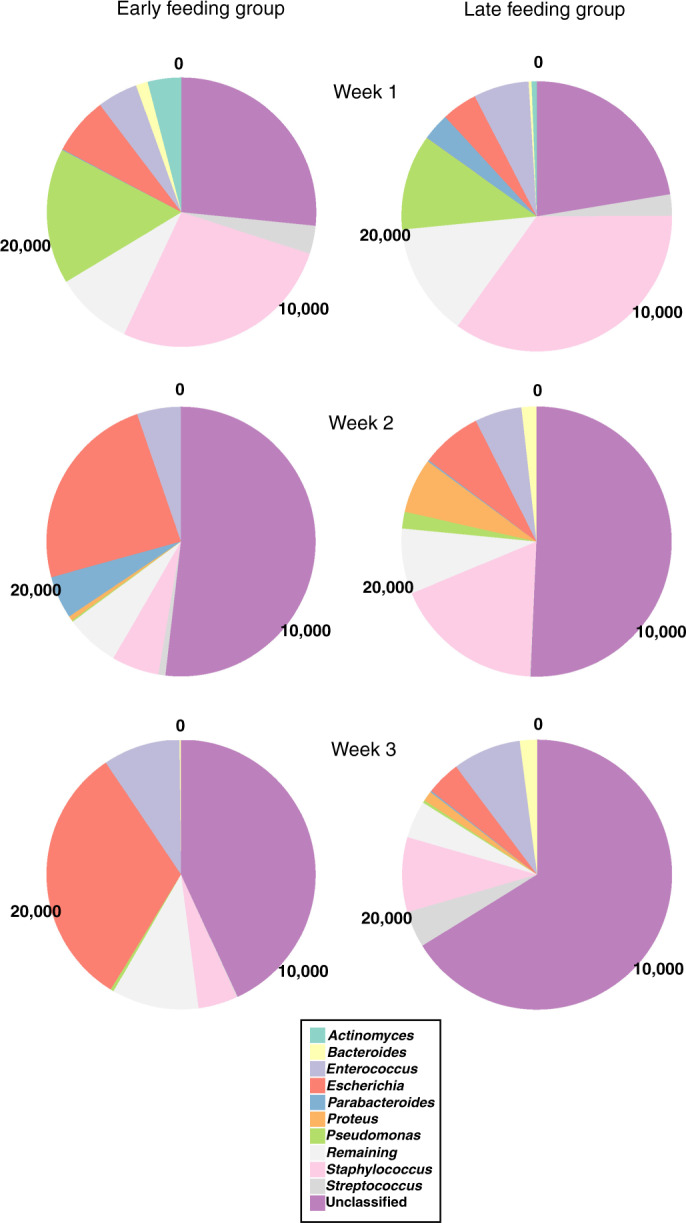
Taxonomic differences at the genus level between groups according to week of life. For the top 10 most common taxa at the genus level, there were no significant differences between groups at week 1 (*n* = 57). At week 2 (*n* = 42), the total read counts of *Staphylococcus* were higher in the late feeding group (*p* = 0.02). During week 3 (*n* = 38), no significant differences in the total read counts were found.

The compositional differences of the gut microbiome at the genus level between groups according to week of life are shown in Fig. 4. The total read counts of *Staphylococcus* during week 2 were higher in the late feeding group. During week 3, the total read counts of *Escherichia* were higher in the early feeding group.

**Fig. 5:**
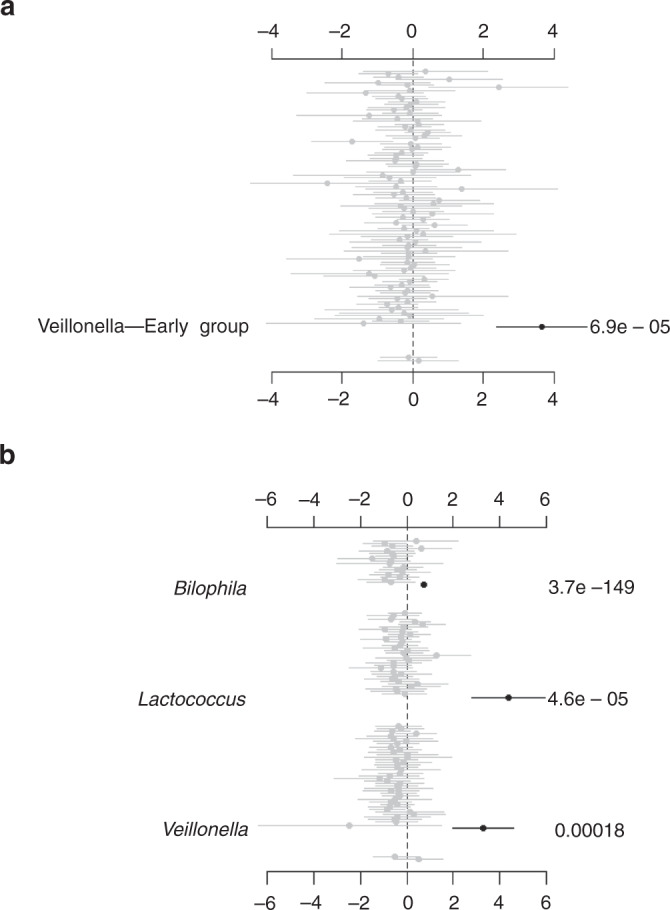
Unadjusted and adjusted longitudinal analysis with negative binomial and zero-inflated mixed models to account for individual variability in the gut microbiome. An unadjusted single-taxon analysis showed that *Veillonella* spp. counts were significantly higher in the early feeding group (**a**). An adjusted single-taxon analysis showed that *Bilophila*, *Veillonella*, and *Lactococcus* spp. were significantly higher in the early feeding group (**b**). The right labels denote statistically significant *p* values.

To explore more subtle single-taxon differences in longitudinal analyses, we first built an NBMM treating the timing of feeding volume progression (i.e., study group) as a fixed effect with a random intercept (Fig. 5a). Using this unadjusted analysis, we identified only the genera *Veillonella* as differentially abundant in the early feeding group. As before, because of the magnitude of the longitudinal changes in intestinal composition with time, we adjusted for other potential covariates. Using this adjusted model, we detected significant differences between the early and delayed feeding groups related to the genera *Veillonella*, *Lactococcus*, and *Bilophila* (Fig. 5b).

Finally, to further explore how microbial communities developed over time in both study groups according to the timing of feeding volume progression, we performed a single-taxon analysis. The absolute count of *Actinomyces*, *Allobaculum*, *Corynebacterium*, *Fusobacterium Lysobacter*, *Methylobacteriaceae*, *Neisseria*, *Peptostreptococcus*, *Planococcaceae*, *Prevotella*, *Pseudomonas*, Rhodospirillaceae, *Staphylococcus*, and *Streptococcus* consistently decreased from week of life 1 to week of life 3. The absolute count of *Proteus*, Enterobacteriaceae, Helicobacteraceae, and Mycoplasmataceae consistently increased from week of life 1 to week of life 3.

## Discussion

With the introduction of enteral feeding after birth, the gut is exposed to the most intense burst of novel antigens in the lifespan.^17^ In this prespecified secondary analysis of a randomized controlled trial of early versus delayed initiation of enteral feeding progression,^8^ we identified three genera of bacteria rigorously associated with the earlier initiation of enteral feeding. In addition, in longitudinal analyses over the first 3 weeks of life, we confirmed temporal differences of the intestinal microbiome in this group. However, the mechanism by which early feeding might specifically favor the growth of these taxa remains unclear.

Our results do not suggest that early life human milk diets during critical periods of development increase the diversity of the gut microbiome in extremely preterm infants. This finding could be related to the small, 3-day difference in the early progression of feeding volumes between groups. Feeding protocols with slower feeding advancement regimens could show different results.

Many observational studies of the intestinal microbiome, and not inconsequentially, many microbiome data analysis pipelines, focus on single observations. However, with this approach, the wide intersubject variability has made drawing reproducible definitions of dysbiosis difficult. As such, it has been recently recognized that longitudinal studies may help overcome this limitation, and indeed large cohorts are beginning to bear this out.^18^ This is particularly true of dynamic processes like the assembly of these complex gut communities. Therefore, in this study in addition to the more traditional static analyses of our cohort, we utilized negative binomial mixed models of longitudinal fecal samples that were collected weekly throughout a dietary intervention. This robust statistical technique allowed us to identify genera whose increased count was associated with early initiation of enteral feeding. While it is beyond the scope of the present study to quantify if these differences are associated with divergent long-term outcomes, they were associated with potentially positive differences in short-term clinical outcomes observed during the randomized clinical trial.^8^

We observed three genera that increased with the early progression of enteral feeding. *Bilophila* species have been shown to bloom with changes in dietary polyunsaturated fatty acids (PUFAs). Interestingly, this has even been shown to apply to maternal diets high in fish oil—while the fetus was in utero. However, at least some members of *Bilophila* are potential pathobionts.^19^ Western diets high in saturated fats, but not PUFAs, promote a sustained increase in the normally low abundance, sulfite-reducing species, *Bilophila wadsworthia*. This change in microbial assemblage was driven by milk-derived-fat-promoted taurine conjugation of hepatic bile acids, which produces an increase in bioavailable sulfur. In *Il10*^*−/−*^ mice, which have an increased susceptibility to colitis, these differences also produced shifts towards a proinflammatory T-helper type 1 immune response that worsened colitis.^20^ While beyond the scope of this study, newborn infants produce significant quantities of bile acids that might bind to breastmilk when it is introduced into the newborn gut. It is worth noting that this does not necessarily indicate that *Bilophila* behaves as a pathobiont in the context of the newborn. Indeed, Bäckhed et al.^21^ observed that *Bilophila* were present in vaginal-born but not cesarean-born infants, suggesting that they were transferred from mother to child. Of note, *Bilophila* among other genera, bloomed with the cessation of breastfeeding and maturation to a more adult microbiome phenotype, further linking these bacteria to changes in diet.

*Veillonella* spp. are gram-negative anaerobic cocci. *Veillonella* are important colonizers of the oral cavity,^22^ where they produce nitrite.^23^ They are also regarded as harmless, potentially beneficial, colonizers of the gastrointestinal and genitourinary tract. Clinically, they show little evidence of pathogenicity. Shifts in the abundance of *Veillonella* have been noticed in structured weight loss programs.^24^ They are known to ferment lactate.^25^ In the context of initial intestinal colonization, their role is currently unknown, but further experimentation should explore if they contribute to the regulation of gut pH during early colonization, as natural competition between *Veillonella* spp. is an important determinant of the composition of the oral microbiome.^22^

*Lactococcus* are lactic acid-producing bacteria that can colonize the gastrointestinal tract. The growth of *Lactococcus lactis* requires the presence of as little as two essential amino acids.^26^ Some *Lactococcus* strains can be isolated from unpasteurized milk. The acidification activity of *Lactococcus lactis* contributes to the breakdown of milk proteins during fermentation and increases resistance to bacteriophage infection.^27^ *Lactococcus lactis* produces antimicrobial enzymes including lacticin that could prevent gram-positive bacterial infections. Early-stage animal studies suggest that *Lactococcus lactis* may be the ideal probiotic to reduce the risk of necrotizing enterocolitis in preterm infants.^28^

A limitation of this study is the resolution provided by 16S-based analyses, which cannot reliably resolve taxonomic identifications down to the species level in many genera. Shotgun metagenomics, under the right conditions, can provide resolution down to the strain level, although at a much higher expense. In addition, 16S-based analyses only detect bacteria and archaea. We have shown that preterm newborns are also significantly colonized by fungi during this period.^29^ Future work could use this higher resolution to perform strain tracking analyses^30^ or real-time reverse transcription-PCR analyses, which could provide more specific information about the assembly of the newborn microbiome. Further exploration of initial intestinal colonization is likely a fertile testbed for the ecological theory of colonization of new environments and may have a significant impact on health. This study could also likely be strengthened by enrolling larger multicenter cohorts of preterm and term-born infants. Another significant limitation of this study is that we did not determine the long-lasting effects of early progression of enteral feeding on the gut microbiome. Our approach to account for exposure to antibiotics and exposure to maternal milk was pragmatic, but it did not capture exposure status at the time of sample collection. Because our study was powered to detect differences in the number of full enteral feeding days, the sample size of this study is small to draw definitive conclusions regarding the gut microbiome. Another limitation is the method used to collect fecal samples. Delays in collecting fecal samples from diapers could have affected the validity of the sequence reads.

In conclusion, we utilized rigorous negative binomial mixed models to perform a longitudinal analysis of multiple extremely preterm infants to account for individual variability in the gut microbiome at three time points set after initiation of enteral feeding. We identified significant differences in three genera that have plausible roles in promoting intestinal health and long-term benefits, but we did not find significant differences in alpha or beta diversity. Future interventional randomized trials and detailed strain tracking using shotgun metagenomics in large cohorts of newborns would help confirm these findings in the future.

## Supplementary information

CONSORT_diagram

FlowDiagram
