# Supplementary material for: The gut microbiome of extremely preterm infants randomized to the early progression of enteral feeding
Source: Pediatr Res. 2021 Nov 13;92(3):799–804. doi: 10.1038/s41390-021-01831-w (PMC9098696; doi:10.1038/s41390-021-01831-w)

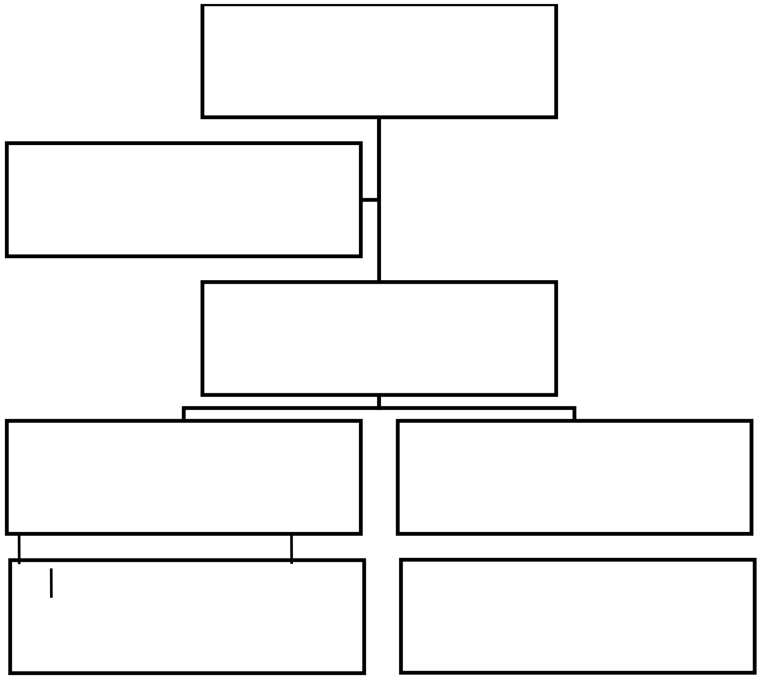

Supplement: Supplementary file 2 [file 41390_2021_1831_MOESM2_ESM.docx]
